# DFST-UNet: Dual-Domain Fusion Swin Transformer U-Net for Image Forgery Localization

**DOI:** 10.3390/e27050535

**Published:** 2025-05-17

**Authors:** Jianhua Yang, Anjun Xie, Tao Mai, Yifang Chen

**Affiliations:** School of Cyber Security, Guangdong Polytechnic Normal University, Guangzhou 510630, China; yangjh86@gpnu.edu.cn (J.Y.); xaj@stu.gpnu.edu.cn (A.X.); thomasmak@stu.gpnu.edu.cn (T.M.)

**Keywords:** image forgery localization, image forensics, dual-domain fusion, swin transformer

## Abstract

Image forgery localization is critical in defending against the malicious manipulation of image content, and is attracting increasing attention worldwide. In this paper, we propose a Dual-domain Fusion Swin Transformer U-Net (DFST-UNet) for image forgery localization. DFST-UNet is built on a U-shaped encoder–decoder architecture. Swin Transformer blocks are integrated into the U-Net architecture to capture long-range context information and perceive forged regions at different scales. Considering the fact that high-frequency forgery information is an essential clue for forgery localization, a dual-stream encoder is proposed to comprehensively expose forgery clues in both the RGB domain and the frequency domain. A novel high-frequency feature extractor module (HFEM) is designed to extract robust high-frequency features. A hierarchical attention fusion module (HAFM) is designed to effectively fuse the dual-domain features. Extensive experimental results demonstrate the superiority of DFST-UNet over the state-of-the-art methods in the task of image forgery localization.

## 1. Introduction

**Figure 1:**
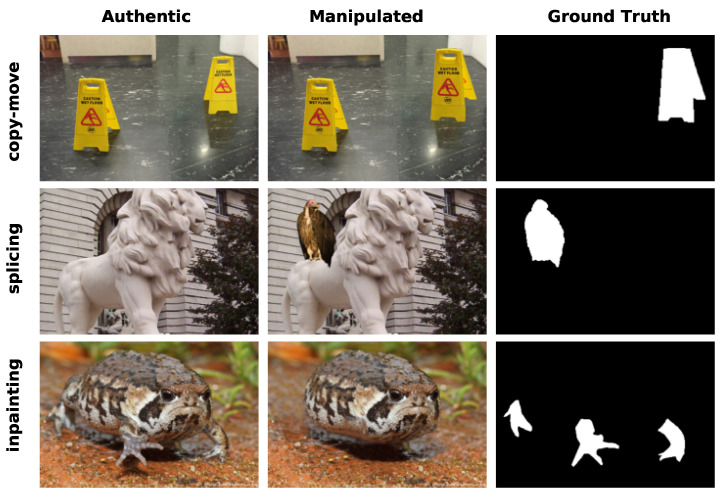
Examples of image forgery with different manipulation techniques. From top to bottom, different tampered techniques such as copy–move, splicing, and inpainting are presented. From left to right, we present the authentic images, manipulated images, and corresponding tampered region masks (ground truth), respectively.

With the availability of powerful image editing software and applications, such as Adobe Photoshop, GIMP, Firefly, etc., users can easily modify images even without any professional knowledge. The most common types of image forgery include copy–move, splicing, and inpainting, as illustrated in Figure 1. Both copy–move and splicing forgery replace one or more regions of the host image (referred to as target regions) with regions of the donor image (referred to as source regions). The difference is that the source regions are from the host image for copy–move forgery, whereas for splicing forgery, the source regions are from other images. Inpainting is a kind of image forgery that eliminates a certain object and repairs the missing regions with the prediction from surrounding pixels.

Although these forgery techniques differ in the process of operation, they could all be used to hide or add meaningful objects in images, and the forged images tend to be indistinguishable from pristine images by the naked human eye. With the widespread use of digital images on the internet, these realistic forged images may be abused by malicious users to create deceptive content and spread false information, leading to severe trust and security concerns. Therefore, it is crucial to develop reliable methods to localize the tampered areas in forged images.

To address this issue, many image forgery localization methods have been proposed in the past. Early works were proposed to explore forgery traces via extracting handcrafted features, such as color filter array (CFA) artifacts [1], noise patterns [2], and compression artifacts [3,4]. However, these traditional methods are time-consuming and require domain knowledge and experience.

In recent years, deep neural networks, such as Convolutional Neural Networks (CNNs), have witnessed significant successes in the field of image forensics due to their strong capability of representation learning. There have been a growing number of CNN-based methods proposed for image forgery localization. Some works were proposed to mainly aim at deploying only one specific image forgery technique, e.g., copy–move [5,6,7,8,9,10], splicing [11,12,13,14,15,16], and inpainting [17,18]. Although these approaches have achieved promising results in specific image forgery, their applicability in practice is limited because various image forgery techniques can be available for a forger, and usually, the type of image forgery techniques used to manipulate images is unknown in real-world scenarios. Therefore, more and more researchers pay attention to general-purpose image forgery localization [19,20,21,22,23,24,25,26,27,28].

Despite advances in CNN-based approaches for image forgery localization, their performance improvements are limited due to the inherent characteristics of CNNs. The intrinsic locality of convolution operations may result in an overemphasis on local features and limitations in modeling long-range dependencies, which reflect the relationships between different regions within an image. However, the long-range dependencies can be used to reveal the inconsistency between the tampered region and the host image, which is an essential clue for image forgery localization.

Recently, self-attention-based architectures, particularly the Transformer [29], have achieved remarkable success in the natural language processing (NLP) domain [30,31] due to their powerful ability to model long-term dependencies in the data. Inspired by the success of Transformers in NLP, researchers have also attempted to introduce Transformers in the field of computer vision. For instance, Vision Transformer (ViT) [32] has emerged as a competitive alternative to CNNs and has been successfully applied to image classification. A growing number of Transformer-based methods have been proposed for image forgery detection and localization [33,34,35,36].

Although most of these methods use ViT or modified ViT as the backbone and outperform CNN-based methods, there are still some concerns: In the vanilla ViT architecture, tokens are all of a fixed scale, while tampered regions are of various scales, which is unsuitable for applications requiring dense prediction at the pixel level, such as image forgery localization. Additionally, the computational complexity of ViT is quadratic in relation to the input image size, making it impractical for high-resolution images. More recently, a new Vision Transformer, called Swin Transformer [37], has been proposed to serve as a general-purpose backbone for computer vision. By adopting a hierarchical architecture and a strategy of the shifted window, Swin Transformer has the flexibility to model at various scales and has linear computational complexity with respect to image size [37].

In this paper, a Dual-domain Fusion Swin Transformer U-Net, denoted as DFST-UNet, is proposed for image forgery localization. DFST-UNet is built on a U-shaped encoder–decoder architecture, which realizes feature fusion between the encoder and decoder by skip connection layers. Swin Transformer blocks are integrated into the U-Net architecture due to their strong capability of modeling long-range dependencies at various scales. Considering the fact that high-frequency forgery information is a critical clue for forgery localization and is difficult to capture from the RGB domain, a dual-stream encoder consisting of the RGB stream and high-frequency stream is proposed to explore forgery feature representations in the RGB domain and frequency domain, respectively. RGB images are fed directly into the RGB stream to learn features related to visual tampering artifacts. In the high-frequency stream, the input images are first processed using a high-frequency feature extractor module (HFEM) to convert the images from the RGB domain to the frequency domain, helping the model to focus on high-frequency-based discriminative features. Instead of adopting SRM filters [19,27] or BayerConv [38] to explore high-frequency information on RGB images, the proposed HFEM is designed to extract more robust high-frequency features by the following successive process: Fast Fourier Transform (FFT), Gaussian high-pass filtering, and Inverse Fast Fourier Transform (IFFT). The two streams of the encoder share the same architecture but have different weights. The dual-domain features are fused using a hierarchical attention fusion module (HAFM), which integrates dual-domain features from the same layer through an attention mechanism.

The main contributions of this paper are highlighted as follows:

- A novel Swin Transformer-based U-shaped architecture is proposed for image forgery localization. Swin Transformer blocks are integrated into the U-Net architecture to capture the long-range context information and identify forged regions at different scales.
- A dual-stream encoder is proposed to comprehensively expose forgery traces in both the RGB domain and the frequency domain. The HAFM is designed to effectively fuse the dual-domain features.
- Experimental results demonstrate that our method outperforms the state-of-the-art (SOTA) methods in image forgery localization, especially regarding generalization and robustness.

The rest of this paper is organized as follows. In Section 2, we review the CNN-based and Transformer-based approaches for image forgery localization. The proposed DFST-UNet is described in detail in Section 3. Experimental results and visualization analysis are reported in Section 4. Finally, the concluding remarks are given in Section 5.

## 2. Related Work

As an important research topic in the field of image forensics, image forgery localization has attracted much attention from researchers over the past decade. Traditional methods were proposed to capture tampering artifacts via extracting handcrafted features, such as color filter array (CFA) artifacts [1], noise patterns [2], and compression artifacts [3,4]. These traditional methods suffer from performance limitations, e.g., generalization ability and robustness. Recently, deep learning-based architectures such as CNNs and Transformers have been utilized to handle the task of image forgery localization due to their powerful feature representation capability and significantly outperformed traditional methods. In this section, we review recent CNN-based and Transformer-based works on general-purpose image forgery localization.

### 2.1. CNN-Based Approaches

Deep neural networks, such as Convolutional Neural Networks (CNNs), have shown promising performance in various computer vision tasks, such as object detection, semantic segmentation, and image classification. However, conventional deep learning frameworks may not be directly applicable to image forgery localization. Unlike conventional computer vision tasks, which primarily focus on image semantic information, image forensics tasks tend to rely on identifying subtle forgery artifacts. Recent CNN-based approaches have been proposed for image forgery localization by specifically designing CNNs to focus on mining critical forgery clues.

Wu et al. [20] formulated the forgery localization problem as a local anomaly detection problem and proposed an end-to-end network named ManTra-Net. A long short-term memory solution was proposed to assess local anomalies. Hu et al. [21] proposed Spatial Pyramid Attention Network (SPAN) for image forgery localization, which models the spatial correlation through a pyramid of local self-attention blocks. Yin et al. [24] proposed a contrastive learning-based multi-task network, which enhances the feature representation by using contrastive learning to measure the consistency of different region statistical properties. Liu et al. [26] developed a Progressive Spatio-Channel Correlation Network (PSCC-Net), which captures the spatial and channel-wise correlations, and estimates manipulation masks at multiple scales. Das et al. [25] proposed a Gated Context Attention Network (GCA-Net), which utilizes non-local attention and a gating mechanism to detect the discrepancy between the authentic and forged regions. Xu [28] proposed a semantic-agnostic progressive subtractive network (SAPS-Net) for image forgery detection and localization. The Semantic-Agnostic Manipulation Attention (SAMA) is designed to reduce the semantic features that serve as manipulation distractors.

Recently, a popular trend to improve the performance of forgery localization is to design a multi-branch structure, which can take both the essential visual and statistical features into consideration, so that rich underlying information can be effectively used. Zhou et al. [19] proposed a two-stream Faster R-CNN network, where one stream extracts RGB features to capture visual tampering artifacts, and the other stream leverages noise features from the steganalysis rich model (SRM). Dong et al. [23] proposed a multi-view feature learning with a multi-scale supervised network (MVSS-Net) for both forgery detection and localization by jointly exploiting edge and noise features. Huang et al. [27] presented a dual-stream UNet named DS-UNet, which includes an RGB stream and a noise stream. The hierarchical fusion is adopted to integrate the features of the two streams to perceive tampered objects at different scales.

Although these CNN-based approaches have achieved promising results, there are still some concerns: In CNN-based approaches, the inherent characteristic of limited receptive fields results in an overemphasis on local features, but limited capability to capture long-range correlation information. However, modeling long-range dependencies contributes to revealing inconsistencies between the tampered and pristine regions, which are critical clues for locating forged regions. Given the strong capability of Transformers in modeling long-range dependencies, in this paper, we explored the Transformer-based architecture to improve image forgery localization performance.

### 2.2. Transformer-Based Approaches

Recently, Transformer-based architectures have been applied to image forgery localization, which can be attributed to their potent ability to model long-range context information.

Hao et al. [33] proposed a novel image forgery localization method, called TransForensics, which is a pioneering work that introduced self-attention mechanisms of Transformers to localize tampered regions. Wang et al. [34] proposed ObjectFormer, a Transformer-based framework for detecting and localizing image manipulations. RGB features and frequency features are combined to identify the tampering artifacts. Learnable object prototypes are leveraged to model visual consistencies within the images. Das et al. [35] proposed Forensic Modulation Network (ForMoNet), which uses focal modulation and window attentions to automatically identify the long- and short-range context for any query pixel. Ma et al. [36] presented a ViT-based model for image manipulation localization (IML), named IML-ViT. A simple feature pyramid network was introduced for conducting multi-scale supervision. A morphology-based edge loss strategy was proposed to ensure edge supervision.

In general, exploring the use of Transformers for image forgery localization is still in its early stages. With advances in Transformers, new ViT variants, such as Swin Transformer [37], have emerged as more suitable for forgery localization due to their flexibility in modeling at various scales. In this paper, we make an attempt to design a Swin Transformer-based architecture for image forgery localization.

## 3. Methodology

In this section, we present the proposed DFST-UNet for image forgery localization. The overall network architecture is described first. Further, the implementation details of the core components are presented.

### 3.1. Overall Network Architecture

**Figure 2:**
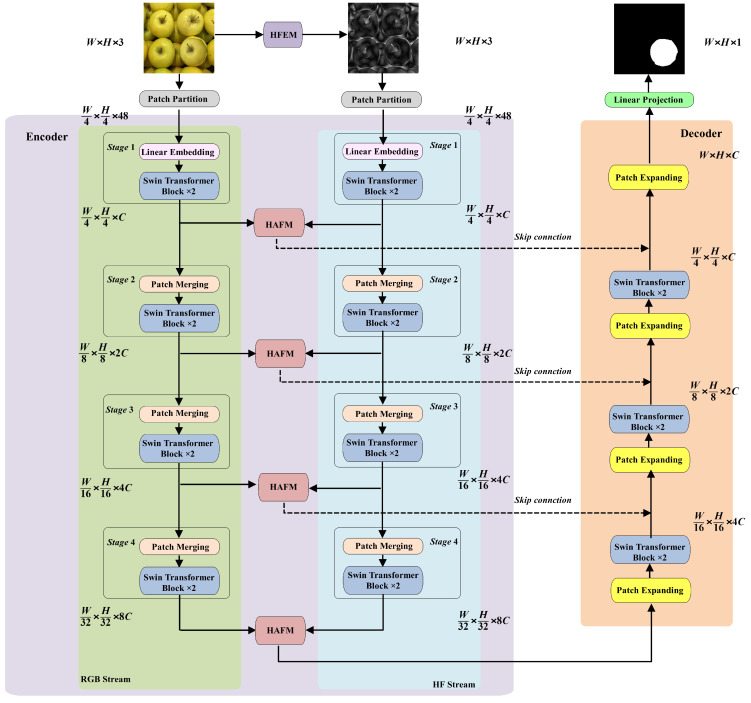
The overall framework of the proposed DFST-UNet.

The overall framework of the proposed DFST-UNet is shown in Figure 2. DFST-UNet adopts the effective U-Net structure, comprising an encoder, a decoder, and skip connections. We employ Swin Transformer blocks as the basic building units because their shifted-window self-attention excels at modeling both local and global dependencies, which is crucial for accurately extracting feature representations and delineating tampered regions. Considering the importance of high-frequency features in image forgery localization—where manipulations often introduce subtle artifacts more visible in the frequency domain—we constructed a dual-stream encoder. In particular, a dual-stream encoder was constructed, consisting of an RGB stream and a high-frequency stream, to explore forgery feature representation in the RGB domain and frequency domain, respectively. RGB images are directly fed into the RGB stream, while for the high-frequency stream, the input images are first converted to the frequency domain by using a high-frequency feature extractor module (HFEM). The two streams share the same architecture but have distinct weights so that they can concentrate on their respective domains. Feature maps of the two streams are fused by a specially designed hierarchical attention fusion module (HAFM) in the corresponding hierarchy. HAFM leverages self-attention to dynamically weigh and integrate complementary cues from the RGB and frequency domains across multiple scales, ensuring that both color–texture and subtle high-frequency artifacts are jointly exploited. Skip connections are adopted to connect the feature maps from the encoder and the decoder.

More formally, an RGB image inputted into the RGB stream is denoted as $X_{s}\in R^{H\times W\times 3}$, where *H* and *W* are the height and width of the image, respectively. The image is converted to the high-frequency feature $X_{h}\in R^{H\times W\times 3}$ by HFEM and used as the input for the high-frequency stream. We split the input RGB image $X_{s}$ into non-overlapping patches of size 4×4 to analogize the “tokens” of sequence data by a patch partition layer. The output is denoted as $P_{s}\in R^{\frac{H}{4}\times \frac{W}{4}\times C}$, where the feature dimension of each patch *C* is 4 × 4 × 3=48. Then, the patch tokens $P_{s}$ are passed to four feature extraction stages, and the output of each stage is defined as $Z_{s}^{\left(n\right)}$, where $n\in \left\{1,2,3,4\right\}$ is the number of the stage. In the stage1, a linear embedding layer is applied to flatten and project these patch tokens into an arbitrary dimension denoted as *C*. We set *C* to 96. The following two Swin Transformer blocks maintain the number of the tokens so that the output $Z_{s}^{\left(1\right)}\in R^{\frac{H}{4}\times \frac{W}{4}\times C}$. In the stage2, a patch-merging layer is applied to produce a hierarchical representation by downsampling and increasing dimension. It concatenates the features of each group of 2×2 neighboring patches, reducing the resolution of the feature maps from $\frac{H}{4}\times \frac{W}{4}$ to $\frac{H}{8}\times \frac{W}{8}$, and the dimensions of the feature maps are 4C. The following linear operations change the dimensions from 4C to 2C. Then, the feature maps are processed by two Swin Transformer blocks to obtain the output $Z_{s}^{\left(2\right)}\in R^{\frac{H}{8}\times \frac{W}{8}\times 2C}$. The same procedure is repeated in stage3 and stage4 to obtain the outputs $Z_{s}^{\left(3\right)}\in R^{\frac{H}{16}\times \frac{W}{16}\times 4C}$ and $Z_{s}^{\left(4\right)}\in R^{\frac{H}{32}\times \frac{W}{32}\times 8C}$, respectively.

In the high-frequency stream, the high-frequency feature $X_{h}$ is passed to the patch partition layer to generate patch tokens $P_{h}\in R^{\frac{H}{4}\times \frac{W}{4}\times C}$, where the feature dimension of each patch C=48. Then, the same processes of four feature extraction stages are performed on $P_{h}$ to obtain $Z_{h}^{\left(n\right)}\in R^{\frac{H}{2^{n+1}}\times \frac{W}{2^{n+1}}\times \left(2^{\left(n-1\right)}C\right)}$, where $n\in \left\{1,2,3,4\right\}$ is the number of the stage. The feature maps of the two streams are extracted from different domains; therefore, they may have significant differences. The proposed HAFM is used to fuse features of each stage from two different domains. The fusion features of stagen can be described by

(1) $$Z_{e}^{\left(n\right)}=Fuse\left(Z_{s}^{\left(n\right)},Z_{h}^{\left(n\right)}\right)$$

where $Z_{e}^{\left(n\right)}\in R^{\frac{H}{2^{n+1}}\times \frac{W}{2^{n+1}}\times \left(2^{\left(n-1\right)}C\right)}$, and $n\in \left\{1,2,3,4\right\}$.

The last fusion feature map $Z_{e}^{\left(4\right)}$ is directly fed into the decoder. And other fusion features $Z_{e}^{\left(n\right)},n\in \left\{1,2,3\right\}$ are connected to the decoder by skip connections in the corresponding hierarchy. The decoder is designed by stacking Swin Transformer blocks and patch-expanding layers. The patch-expanding layer performs feature upsampling by reshaping feature maps of adjacent dimensions into a large feature map with 2× upsampling of the resolution. In the end, the last patch-expanding layer is used to perform 4× upsampling to restore the resolution of the feature maps, so that we obtain the final output of the encoder as $Z_{d}\in R^{H\times W\times C}$. Finally, a linear projection layer is applied on $Z_{d}$ to produce a predicted mask with only one channel of the same size as the input image, representing the possibility of the authentic and forged pixel.

### 3.2. High-Frequency Feature Extractor

High-frequency features are critical for image forgery localization, as manipulation operations often introduce subtle artifacts along object boundaries and splice seams—artifacts that are difficult to detect in the RGB domain but become more prominent in high-frequency components. High-frequency information can be extracted either in the spatial domain or the frequency domain. Spatial-domain methods focus on pixel-level residuals and edge enhancement.

We designed a high-frequency feature extractor in the frequency domain to extract robust high-frequency features. An input image $X_{s}\in R^{H\times W\times 3}$ is split into three color channels, i.e., *R*, *G*, and *B*. For the $k_{th}$ color channel $X_{s}^{k}\left(x,y\right)$ of size *H* × *W*, the process of extracting high-frequency features is as follows: First, $X_{s}^{k}\left(x,y\right)$ is transformed from the spatial domain to the frequency domain by using Fast Fourier Transform (FFT). It can be denoted as

(2) $$F^{k}\left(u,v\right)=\sum_{x=0}^{H-1}\sum_{y=0}^{W-1}X_{s}^{k}\left(x,y\right)\cdot e^{-2\pi i\left(\frac{ux}{H}+\frac{vy}{W}\right)}$$

where $\left(x,y\right)$ and $\left(u,v\right)$ are the coordinates of the image in the spatial domain and frequency domain, respectively. Second, a Gaussian high-pass filter (GHPF) formulated as Equation (3) is applied to the image for filtering in the frequency domain:

(3) $$H\left(u,v\right)=1-e^{-\frac{D^{2}\left(u,v\right)}{{2D_{0}}^{2}}}$$

where $D_{0}\in R$ is the cut-off frequency, which is set to 30 in our implementation, and $D\left(u,v\right)$ is the distance from the frequency point $\left(u,v\right)$ to the center of the spectrum. The filtering result can be noted as

(4) $$G^{k}\left(u,v\right)=F^{k}\left(u,v\right)\cdot H\left(u,v\right)$$

Finally, the frequency domain information is transformed back to the spatial domain information by using Inverse Fast Fourier Transform (IFFT). It can be denoted as

(5) $$X_{h}^{k}\left(x,y\right)=\frac{1}{HW}\sum_{u=0}^{H-1}\sum_{v=0}^{W-1}G^{k}\left(u,v\right)\cdot e^{2\pi i\left(\frac{ux}{H}+\frac{vy}{W}\right)}$$

The final output $X_{h}\in R^{H\times W\times 3}$ is obtained by extracting the high-frequency features from each color channel.

In the ablation study (Section 4.5) and the high-frequency feature visualization (Section 4.4), we evaluated several high-frequency feature extraction methods. The results demonstrate that our proposed method achieves superior performance in image forgery localization, confirming its effectiveness in robust high-frequency modeling.

### 3.3. Hierarchical Attention Fusion Module

The two streams of the encoder were designed to learn the forgery feature representations from the RGB domain and frequency domain, respectively. As a result, the feature maps extracted from the two streams may be significantly different. To address this within the multi-scale framework of the proposed U-shaped network, we designed a hierarchical attention fusion module (HAFM) for efficient fusion of multi-scale features from two different streams.

**Figure 3:**
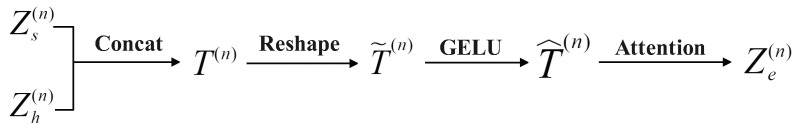
Fusion process of HAFM.

The computational process of the fusion feature of the $n_{th}$ stage is illustrated in Figure 3. The outputs of the $n_{th}$ stage in the RGB stream and the high-frequency stream, i.e., $Z_{s}^{\left(n\right)}\in R^{\frac{H}{2^{n+1}}\times \frac{W}{2^{n+1}}\times 2^{\left(n-1\right)}C}$ and $Z_{h}^{\left(n\right)}\in R^{\frac{H}{2^{n+1}}\times \frac{W}{2^{n+1}}\times 2^{\left(n-1\right)}C}$ are first concatenated along the channel dimension to obtain $T^{\left(n\right)}\in R^{h\times w\times 2c}$. Here, $h=\frac{H}{2^{n+1}}$, $w=\frac{W}{2^{n+1}}$, and $c=2^{n-1}C$. Concatenating features from the RGB and high-frequency streams enables the integration of complementary information: RGB features provide color and texture, while high-frequency features reveal subtle manipulation artifacts and edges, leading to more comprehensive forgery cues. Then, $T^{\left(n\right)}$ is reshaped to ${\tilde{T}}^{\left(n\right)}\in R^{h\times w\times c}$ by performing a linear layer. The linear layer compresses the concatenated features back to the original channel size, maintaining a balance between information integration and computational efficiency, while avoiding redundancy. ${\tilde{T}}^{\left(n\right)}$ is processed by the GELU activation function to obtain ${\hat{T}}^{\left(n\right)}$, where GELU enhances the model’s nonlinear representation capabilities. Finally, we conducted the self-attention (SA) operation [29], followed by a residual connection and normalization, to obtain the output of the HAFM of the *n*th stage $Z_{e}^{\left(n\right)}\in R^{\frac{H}{2^{n+1}}\times \frac{W}{2^{n+1}}\times 2^{\left(n-1\right)}C}$. The self-attention mechanism dynamically weighs the contributions of different regions and modalities, enabling precise fusion of critical features, especially around manipulated boundaries. Residual connections preserve gradient flow, facilitating stable deep feature learning, while LayerNorm accelerates convergence and mitigates feature distribution shifts, thus improving robustness, which can be formulated as

(6) $$\begin{array}{cc} \; & \mathrm{SA}\left({\hat{T}}^{\left(n\right)}\right)=\mathrm{softmax}\left(\frac{Q^{\left(n\right)}(K^{\left(n\right)}{)}^{T}}{\sqrt{L}}\right)V^{\left(n\right)}\\ \; & Z_{e}^{\left(n\right)}=\mathrm{Norm}\left(\alpha \cdot \left(\mathrm{SA}({\hat{T}}^{\left(n\right)})\right)+{\hat{T}}^{\left(n\right)}\right) \end{array}$$

where $Q^{\left(n\right)}$, $K^{\left(n\right)}$, and $V^{\left(n\right)}$ are the query, key, and value matrices, respectively, which can be computed as $Q^{\left(n\right)}={\hat{T}}^{\left(n\right)}W_{q}$, $K^{\left(n\right)}={\hat{T}}^{\left(n\right)}W_{k}$, and $V^{\left(n\right)}={\hat{T}}^{\left(n\right)}W_{v}$. Here, $W_{q}$, $W_{k}$, and $W_{v}$ are the learnable parameters of three linear projection layers in self-attention. The parameter α is a learnable weight parameter, and Norm denotes LayerNorm.

### 3.4. Swin Transformer Block

The conventional Transformer block consists of a multi-head self-attention (MSA), a multi-layer perceptron (MLP), and layer normalization (LN). The global self-attention among all tokens is calculated in MSA, leading to the quadratic computational complexity of the number of tokens. For more efficient modeling, the Swin Transformer [37] is built by replacing the standard MSA module in each Transformer block with a module based on shifted windows, with other layers kept the same. The window-based MSA with two partitioning configurations, namely regular window configuration (W-MSA) and shifted window configuration (SW-MSA), is introduced to divide the image into non-overlapping windows and perform self-attention computation within the windows, where each window only covers M×M patches. W-MSA and SW-MSA are applied alternately in consecutive Swin Transformer blocks to enhance the information connection across windows.

**Figure 4:**
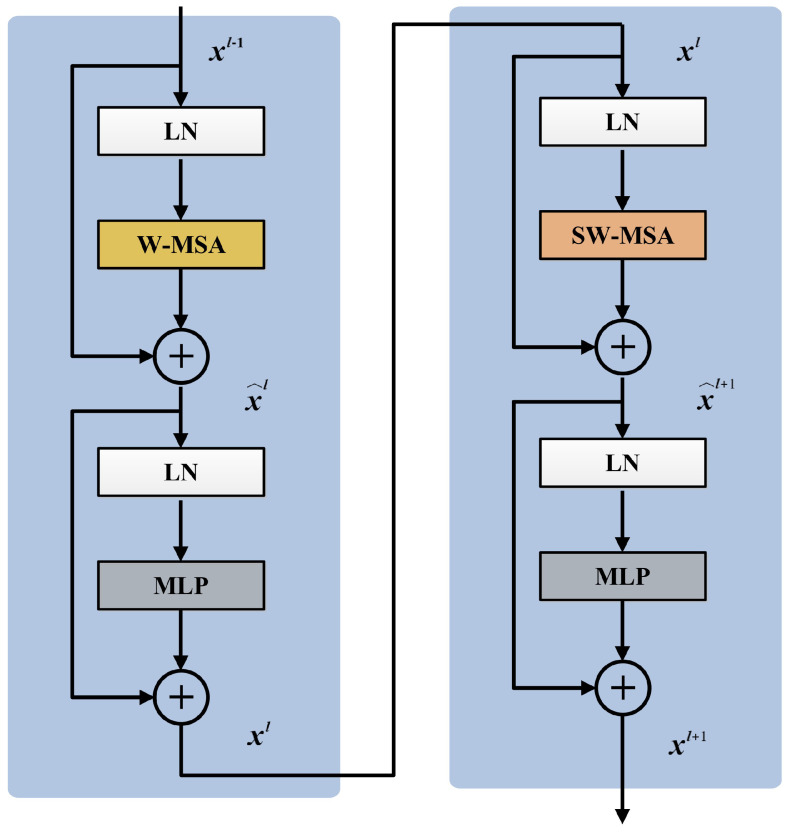
Swin Transformer block.

Figure 4 shows two consecutive Swin Transformer blocks. Based on the shifted window partitioning mechanism, continuous Swin Transformer blocks can be formulated as

(7) $$\begin{array}{cc} \; & {\hat{x}}^{l}=W\text{-}\mathrm{MSA}(\mathrm{LN}\left(x^{l-1}\right))+x^{l-1}\\ \; & x^{l}=\mathrm{MLP}(\mathrm{LN}\left({\hat{x}}^{l}\right))+{\hat{x}}^{l}\\ \; & {\hat{x}}^{l+1}=\mathrm{SW}\text{-}\mathrm{MSA}(\mathrm{LN}\left(x^{l}\right))+x^{l}\\ \; & x^{l+1}=\mathrm{MLP}(\mathrm{LN}\left({\hat{x}}^{l+1}\right))+{\hat{x}}^{l+1} \end{array}$$

where ${\hat{x}}^{l}$ and ${\hat{x}}^{l+1}$ represent the output of W-MSA and SW-MSA, respectively. $x^{l}$ and $x^{l+1}$ represent the outputs after passing through the MLP layer. In the MLP layer, dimension alteration and nonlinear transformation are performed on each token, thereby enhancing the representation ability of tokens. LN is applied before each sub-layer, with a residual connection surrounding them.

### 3.5. Loss Function

We employed a combination of the binary cross-entropy loss ($L_{bce}$) and Dice loss ($L_{dice}$) to train the proposed DFST-UNet. To be specific, the predicted localization mask *P* is supervised by the ground truth mask *G* using the pixel-scale binary cross-entropy loss, which is defined as follows:

(8) $$\begin{array}{c} L_{bce}\left(P,G\right)=-\frac{1}{HW}\sum_{i=1}^{H}\sum_{j=1}^{W}\left(G\left(i,j\right)logP\left(i,j\right)+\left(1-G\left(i,j\right)\right)log\left(1-P\left(i,j\right)\right)\right) \end{array}$$

where G(i,j) and P(i,j) denote the ground truth labels and the manipulated image predictions of the pixel at location (i,j) in an image.

Moreover, considering the fact that the area of the manipulated regions is generally much smaller than that of the pristine regions, we introduced the Dice loss [39], which has proven to be effective for learning from extremely imbalanced data. The Dice loss can be expressed as

(9) $$\begin{array}{c} L_{dice}\left(P,G\right)=1-\frac{2\left|P\cap G\right|}{\left|P\right|+\left|G\right|+\epsilon } \end{array}$$

where the hyper-parameter is set as $\epsilon =10^{-6}$ to prevent zero-division.

The overall loss function *L* of the proposed model can be described as follows:

(10) $$L=\lambda L_{bce}\left(P,G\right)+\left(1-\lambda \right)L_{dice}\left(P,G\right)$$

where the trade-off coefficient λ is set to 0.9. This value was selected based on empirical experiments; we tested multiple weight combinations and found that excessively high Dice loss weights caused the model to fail to converge.

## 4. Experiment

In this section, we present the extensive experiments conducted to demonstrate the effectiveness of our proposed DFST-UNet for image forgery localization. Our method is compared with several SOTA methods on public datasets.

### 4.1. Experimental Setup

#### 4.1.1. Dataset

We conducted experiments on five public datasets, including Columbia [40], COVERAGE [41], CASIA (v1 + v2) [42], NIST16 [43], and IMD2020 [44]. The details of these datasets are provided in Table 1. Columbia [40] consists of 180 spliced images, with sizes ranging from 757 × 568 to 1152 × 568. Specifically, the tampered images in Columbia are uncompressed and have undergone no post-processing. COVERAGE [41] comprises 100 copy–move tampered images. CASIA [42] includes two versions, v1 and v2. CASIA-v1 and CASIA-v2 contain 920 and 5123 tampered images, respectively, and both involve two types of manipulations, i.e., copy–moving and splicing. NIST16 [43] includes 564 ultra-high-resolution tampered images with an average resolution of 3460 × 2616, involving three manipulations, i.e., splicing, copy–move, and inpainting. IMD2020 [44] comprises 2010 real-world tampered images collected from the internet, involving all three manipulations.

#### 4.1.2. Evaluation Metrics

To quantify localization performance, we followed the previous work [26] by adopting pixel-level Area Under the Curve (AUC) and F1 score between the ground truth (GT) and predicted masks as our evaluation metrics. AUC indicates the relationship between the True Positive Rate (TPR) and False Positive Rate (FPR) at different thresholds. F1 score is the harmonic mean of Precision and Recall. When calculating the F1 scores, the threshold for binary masks and detection scores is set to 0.5. Both the pixel-level AUC and F1 score values range in [0, 1], with values closer to 1 indicating better performance.

#### 4.1.3. Implementation Details

All experiments were carried out using the PyTorch 1.12.0 framework on a GeForce RTX 4090 GPU. The backbone network was initialized with pre-trained weights of Swin-Unet. The network was trained using the AdamW optimizer with a weight decay of 0.05, an initial learning rate of 1.25 $\times \;10^{-4}$, a minimum learning rate of 1.25 $\times \;10^{-7}$, and a cosine annealing learning rate scheduler. A linear warm-up strategy was applied to the learning rate for the first 5 epochs. The training batch size was set to 48, and all input images were resized to 224. We performed data augmentation on the training set in a random manner, including random rotations (0°, 90°, 180°, 270°) and random flips (horizontal, vertical).

### 4.2. Comparisons with SOTA Methods

The compared image forgery localization methods can be divided into two categories: (1) CNN-based approaches such as RGB-N [19], ManTra-Net [20], SPAN [21], MVSS++ [23], PSCC [26], DS-UNet [27], and DPMSN [45]; and (2) Transformer-based methods such as TransForensics [33], ObjectFormer [34], and ForMoNet [35]. Following previous works, we compared localization performance under two different experimental scenarios: (1) Pre-training: The model is trained on the publicly available CASIA-v2 dataset and then tested on four other public datasets individually. The pristine images from CASIA-v2 (a total of 7295 images) are also used during training. (2) Fine-tuning: The pre-trained models are further fine-tuned on the training splits of COVERAGE, NIST16, and IMD2020, respectively, and then evaluated on their corresponding test split. The train/test splits for pre-training and fine-tuning are detailed in Table 2.

#### 4.2.1. Pre-Training Scenario

We evaluated the generalization capability of the proposed DFST-UNet on four public image forgery datasets and compared it with SOTA methods, including ManTra-Net [20], SPAN [21], MVSS++ [23], PSCC [26], DS-UNet [27], TransForensics [33], ObjectFormer [34], and ForMoNet [35]. The reported results of PSCC [26], DS-UNet [27], and ObjectFormer [34] were obtained by training the models with their official public codes on our training samples, while other results were sourced directly from their respective papers. Table 3 summarizes the comparison results of generalization performance under the AUC metric. It can be observed that the pre-trained DFST-UNet achieves the best average AUC result, which indicates superior generalization overall. The proposed DFST-UNet achieves the best localization performance on Columbia [40] and IMD2020 [44], and ranks third on COVERAGE [41] and second on NIST16 [43]. The reason that SPAN [21] surpasses DFST-UNet on COVERAGE [41] might be the differences in training datasets. The CASIA-v2 dataset we used contains only 3295 copy–moved images, whereas SPAN [21] was trained on the synthetic dataset consisting of 100 k copy-moved images. It seems that the training dataset of SPAN [21] has a more similar data distribution to COVERAGE [41], which is a small dataset focusing on copy–move. Although our training dataset (i.e., CASIA-v2) only involves two manipulations, namely splicing and copy–move, the proposed model shows comparable performance to the best method (i.e., ForMoNet [35]) when testing on NIST16 [43], which contains unknown manipulated images, such as inpainting images.

We conducted a fair comparison with PSCC * [26], DS-Unet * [27], and Objectformer * [34] by training on the same dataset. Our model achieved the best performance on all four datasets. When testing on the IMD2020 [44] dataset, which is a realistic challenge dataset, our model obtains performance improvements of the AUC scores with gains of 0.7%, 1.3%, and 3.3% when compared with PSCC * [26], DS-Unet * [27], and Objectformer * [34], respectively. It indicates the better generalization of our method when handling real-world cases. Moreover, the proposed model improves the average accuracy by 5.95% than Objectformer * [34], which is a SOTA Transformer-based method.

#### 4.2.2. Fine-Tuning Scenario

We further fine-tune the pre-trained models on the training split of COVERAGE [41], NIST16 [43], and IMD2020 [44] datasets, respectively. The best models are selected to test on the corresponding datasets. The quantitative comparison of the AUC and F1 scores under the fine-tuning experimental setup is reported in Table 4. It is observed that our proposed method ranks the best with regard to performance on each dataset in terms of AUC scores. In particular, the performance of DFST-UNet on COVERAGE [41] surpasses SPAN [21] after fine-tuning, demonstrating the ability of the proposed model to learn forgery-related features adaptively. Furthermore, DFST-UNet outperforms Objectformer * [34] with average AUC and F1 score improvements of 9.77% and 51.36%, respectively. This validates the effectiveness of the proposed Transformer-based architecture.

### 4.3. Visualization of Model Prediction Masks

**Figure 5:**
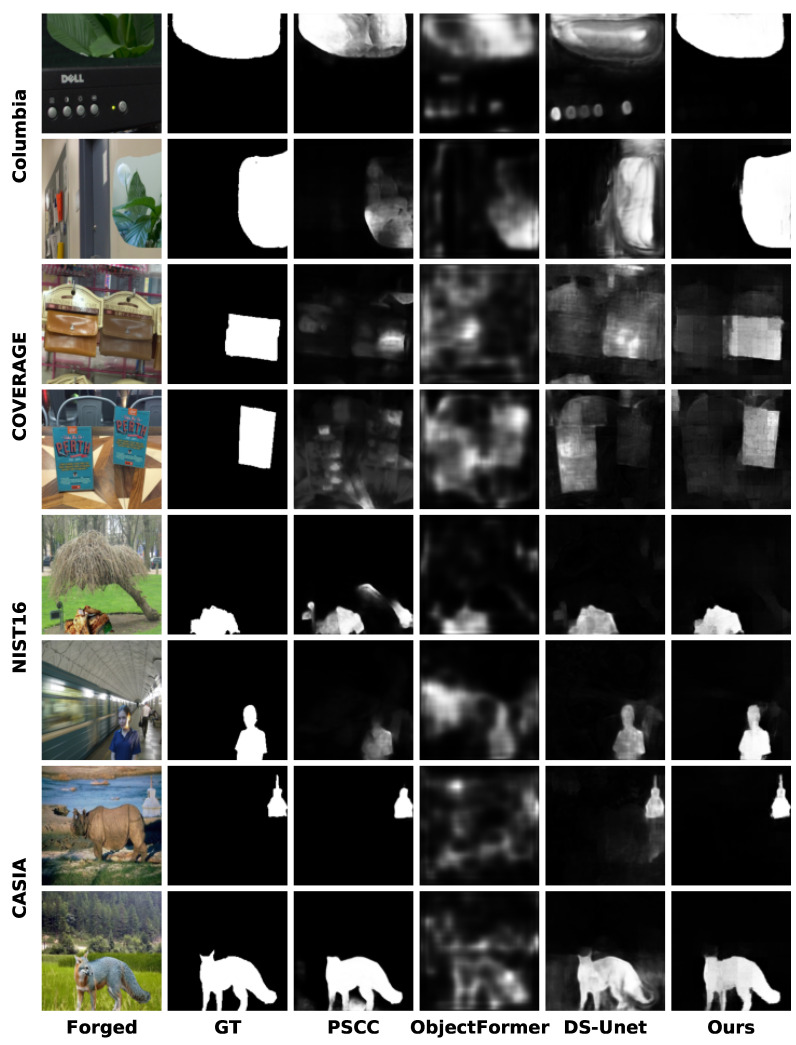
Visualizations of the predicted tampering localization masks under the pre-trained scenario for different methods on the Columbia [40], COVERAGE [41], CASIA [42], and NIST16 [43] datasets. From left to right, the images shown are the forged image, GT mask, PSCC [26], ObjectFormer [34], DS-UNet [27], and our prediction.

We visualize the predicted masks generated by our model and compare them with SOTA methods including PSCC [26], ObjectFormer [34], and DS-UNet [27] under the pre-training scenario. Figure 5 presents the comparative results for the images from Columbia [40], COVERAGE [41], NIST16 [43], and CASIA [42]. It can be seen that the proposed DFST-UNet can provide more accurate manipulated region predictions, and the localization boundaries of tampered regions are finer compared to other SOTA methods. With Swin Transformer blocks integrated into the DFST-UNet, long-range context information reflecting the relationships between different regions within an image can be captured at various scales, resulting in satisfying predictions closer to the ground truth. Additionally, high-frequency features are exploited to identify subtle forgery artifacts, enhancing boundary detection precision.

**Figure 6:**
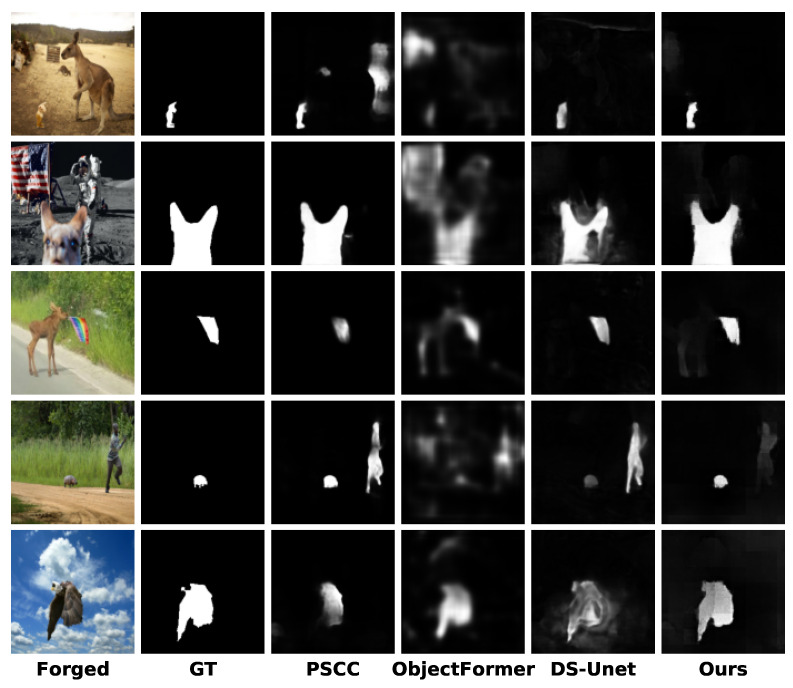
Visualizations of the predicted tampering localization masks under the pre-trained scenario for different methods on real-world tampering dataset IMD2020 [44].

Figure 6 shows the comparisons on the real-world tampering dataset IMD2020 [44]. This dataset provides realistic images that are collected from the internet and are carefully selected to remove obvious manipulation traces. Therefore, the evaluation of IMD2020 is more representative of real-world application scenarios. The results demonstrate that the proposed DFST-UNet can locate the forgery areas with high accuracy at different scales. As seen in the fourth row, DFST-UNet can recognize the manipulation item with fewer false alarms, even when the area of the region is extremely small. In the first row, despite the low contrast between the forged region and the surrounding background, our method can exclude the similarity interference of the pristine region in the background and successfully localize the forged region. These comparison results further highlight the superior performance of the proposed DFST-UNet in image forgery localization, especially in practical scenarios.

**Figure 7:**
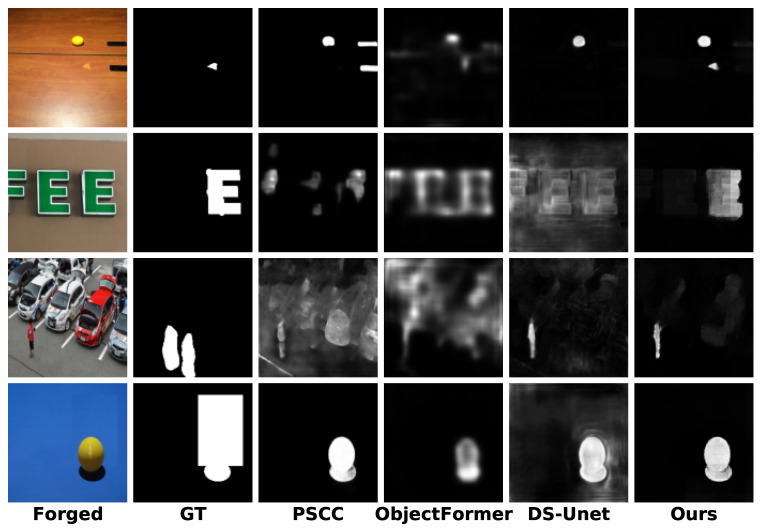
Visualizations of failure cases or limitations of different methods under the pre-trained scenario.

Although our method performs well overall, there are still limitations and errors in certain specific scenarios, as shown in Figure 7. Specifically, in the first row, the yellow ball is mistakenly identified as a tampered region; in the second row, the predicted mask exhibits blurry and imprecise boundaries; in the third row, the restored area is highly similar to the original background, leading to the misclassification of untampered regions; and in the fourth row, the model fails to suppress background interference, resulting in incomplete localization of the tampered area. A key contributing factor to these issues is the uniform resizing of input images to 224 × 224 resolution. While this improves computational efficiency and training stability, it inevitably leads to the loss of fine-grained visual features, particularly when detecting small-scale or boundary-blurred manipulations. We plan to address these challenges in two directions in the future: first, by introducing multi-scale edge supervision mechanisms to improve boundary localization; and second, by employing more effective preprocessing strategies such as overlapping patch division, which helps preserve key features in tampered regions and enhances detection performance under complex real-world conditions.

### 4.4. Robustness Analysis

To evaluate the robustness of the proposed method, we follow the distortion settings in the previous works [21,26,34] to degrade the quality of the original images in the NIST16 [43] dataset. These distortions include resizing with different scaling factors (0.78×, 0.25×), Gaussian blur with the kernel size k∈{3, 15}, Gaussian noise with the standard deviation σ∈{3, 15}, and JPEG compression with the quality factor q∈{100, 50}. Table 5 presents a robustness comparison based on AUC scores for our pre-trained model and other SOTA methods, including ManTra [20], SPAN [21], PSCC * [26], ObjectFormer * [34], and DS-UNet * [27]. It can be seen that our model outperforms the others under the distortion operations of resizing, Gaussian noise, and JPEG compression, indicating superior robustness against these distortions. The performances of our model rank second under Gaussian blur and are slightly lower than those of DS-UNet *. This may be because we adopt Gaussian high-pass filtering in the HFEM to dig into the subtle forgery artifacts in the frequency domain.

**Figure 8:**
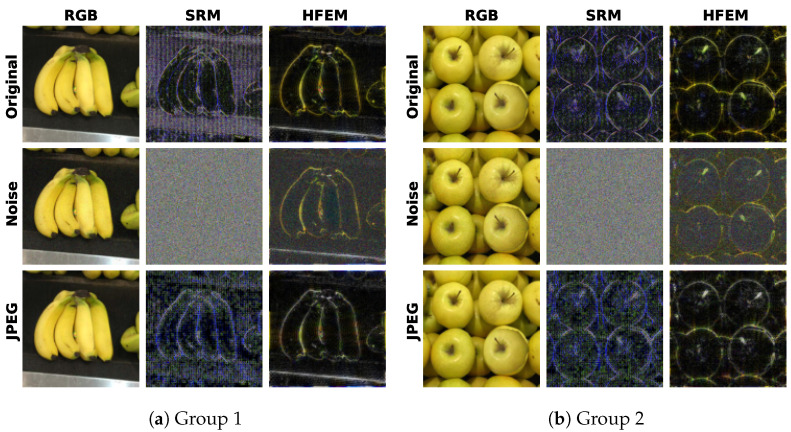
Visualizations of two groups of high-frequency features. In each group, the original RGB image, the image post-processed by Gaussian noise (σ = 15), and JPEG compression (q = 50) are shown from top to bottom. For each image, we show its high-frequency features extracted by SRM filters and HFEM, respectively.

The satisfactory robustness can be attributed to the robust feature learning of the proposed HFEM. In Figure 8, we show the high-frequency features obtained from original and post-processed images by SRM filters used in previous works [19,20,27] and the proposed HFEM, respectively. It can be seen that the features extracted by SRM filters are more sensitive to distortion operations, while the proposed HFEM is less affected by distortion operations and exhibits more robust performance.

### 4.5. Ablation Analysis

To validate the effectiveness of our network design, we construct the variants of DFST-UNet with different branch structures, high-frequency extraction methods, and feature fusion methods and evaluate their performance on the Columbia [40] dataset under a pre-training scenario. The setup variants are as follows:

- Baseline: We use Swin-Unet [46] as the baseline model with only RGB images as input.
- Model-v1: This model is constructed by replacing the HFEM of DFST-UNet with the SRM filters.
- Model-v2: This model is constructed by replacing the HFEM of DFST-UNet with the Bayar convolutions [47].
- Model-v3: This model is constructed on the basis of the baseline. Instead of inputting RGB images, the proposed HFEM is adopted to generate high-frequency features as the input.
- Model-v4: This model is constructed by replacing the HAFM of DFST-UNet with the element-wise addition.
- Model-v5: This model is constructed by replacing the Gaussian high-pass filter in HFEM with an ideal high-pass filter, and retaining the HAFM for feature fusion.

Table 6 presents the ablation analysis under the metrics of AUC and F1 score. We can observe that our proposed components are all effective and improve AUC and F1 scores significantly. Firstly, we investigated the influence of different branch structures on performance by comparing the Baseline, Model-v3, and the proposed DFST-UNet. It is observed that adopting only RGB images or high-frequency features cannot achieve the optimum localization performance. The dual-stream structure of DFST-UNet can comprehensively capture forgery artifacts in both the RGB domain and the frequency domain, therefore achieving superior performance. Moreover, comparing Model-v3 with the baseline shows that using only the proposed HFEM to generate high-frequency features improves the AUC from 91.82% to 93.43% and the F1 score from 45.76% to 59.51%. This demonstrates the superiority of the proposed HFEM, which effectively suppresses redundant low-frequency information and enhances manipulation traces such as edge inconsistencies and unnatural transitions.

Then, we explored different high-frequency extraction methods by replacing the HFEM of DFST-UNet with the SRM filters (Model-v1) and Bayar convolutions (Model-v2), respectively. The SRM filters used in Model-v1 are traditional handcrafted filters widely used in image forensics, as adopted in DS-UNet [27]. The Bayar convolutions used in Model-v2 are constrained convolutional layers employed in MVSS [23] to suppress semantic content and highlight manipulation cues. The comparison results show that DFST-UNet improves the AUC by 7.67% and F1 score by 41.73% compared with Model-v1. In addition, DFST-UNet outperforms Model-v2 with 6.96% and 28.52% improvements in AUC and F1 scores, respectively. The reason for the superior performance of our designed HFEM might be that HFEM extracts high-frequency features in the frequency domain rather than in the spatial domain, which can more effectively capture the frequency-related inconsistency between the manipulated region and the pristine region. Moreover, we further compare our HFEM with a variant using an ideal high-pass filter (Model-v5) instead of the Gaussian high-pass filter. The proposed method surpasses Model-v5 by 0.05% in AUC and 0.34% in F1 score. This confirms that the Gaussian high-pass filter, by offering a smoother frequency response and preserving edge continuity, enables more stable and discriminative high-frequency feature extraction, thereby enhancing localization performance.

Finally, we analyze the effect of different fusion schemes between the RGB and the high-frequency stream. Model-v4 employs element-wise addition as the fusion method, which is also the strategy adopted in DS-UNet [27]. Compared with Model-v4, our method achieves significant performance gain with AUC and F1 score improvements of 4.01% and 20.44%, respectively. It verifies the effectiveness of the proposed HAFM. The feature representations learned from the RGB stream and high-frequency stream may be significantly different. Fusing the dual-domain features by element-wise addition may incur instability. Furthermore, although Model-v4 incorporates both RGB and high-frequency features, its performance still drops compared to Model-v3, with decreases of 1.85% in AUC and 13.27% in F1 score. This suggests that directly adding HFEM-extracted high-frequency features to the RGB features element-wise may cause the model to assign identical initial weights to both tampered and untampered regions, thus weakening its discrimination ability. In contrast, HAFM based on the self-attention mechanism can flexibly focus on the important relationships between different features, achieving more effective feature fusion. These results further demonstrate the superiority of our proposed HAFM in handling cross-domain feature integration.

Overall, all the proposed components demonstrate clear effectiveness. Incorporating only the HFEM on top of the Swin Transformer backbone (Model-v3) leads to improvements of 1.61% in AUC and 13.75% in F1 score compared to the baseline, validating its ability to enhance manipulation-related features. Furthermore, replacing the element-wise addition fusion strategy in Model-v4 with the proposed HAFM yields further gains of 4.01% in AUC and 20.44% in F1 score. These results indicate that HAFM contributes the most among the proposed modules, as it facilitates more stable and discriminative cross-domain feature integration.

### 4.6. Model Complexity and Computational Efficiency Analysis

To comprehensively evaluate the practicality and efficiency of our proposed DFST-UNet, we compare it with several SOTA methods, including PSCC [26], DS-UNet [27], and ObjectFormer [34], in terms of model complexity and computational efficiency. The evaluation metrics include GFLOPs (Giga Floating Point Operations), parameter size (in millions), maximum memory usage (in MB), and average inference time (in milliseconds). All statistics are estimated using an input resolution of (3,224,224) on a GeForce RTX 4090 GPU. The results are summarized in Table 7. The best results for each metric are highlighted in bold to facilitate intuitive comparison.

Our DFST-UNet demonstrates an excellent trade-off between detection performance and computational efficiency. It requires only 11.95 GFLOPs, which is significantly lower than ObjectFormer [34] (65.20), PSCC [26] (37.45), and DS-UNet [27] (23.06). In terms of parameter count, DFST-UNet (54.46M) is larger than the CNN-based PSCC [26] (2.75 M) and DS-UNet [27] (40.30 M), but substantially smaller than the Transformer-based ObjectFormer [34] (130.57 M). This demonstrates that DFST-UNet achieves an efficient architectural design with moderate complexity.

Although PSCC [26] has a small model size, it consumes the most GPU memory (1905 MB), likely due to its HRNet backbone, which preserves high-resolution feature maps throughout the network and leads to significant memory overhead. In contrast, DFST-UNet maintains a moderate memory footprint (1200 MB) and inference time (21.86 ms), comparable to PSCC [26] and faster than DS-UNet [27]. Despite being based on the Swin Transformer, DFST-UNet shares the same U-Net-style encoder–decoder architecture as DS-UNet [27], involving extensive downsampling, upsampling, and feature concatenation across multiple scales. These operations, which are also present in PSCC [26], are computationally expensive and less GPU-efficient compared to the simpler, more streamlined inference path in ObjectFormer [34], which avoids such hierarchical operations. This explains why, despite DFST-UNet’s lower GFLOPs, it has slightly higher inference time compared to ObjectFormer [34].

Overall, DFST-UNet achieves lower computational cost and balanced model complexity, indicating strong generalization ability and efficient inference. These characteristics make it a promising choice for real-world applications, where both accuracy and speed are essential.

## 5. Conclusions

In this work, we propose a novel DFST-UNet for image forgery localization, which is built by integrating Swin Transformer blocks into a U-shaped encoder–decoder architecture. We introduce a dual-stream encoder to comprehensively explore tampering traces in both the RGB and frequency domains. Additionally, we design a hierarchical attention fusion module (HAFM) to effectively fuse the dual-domain features. Extensive experiments demonstrate that our DFST-UNet outperforms SOTA methods in image forgery localization. Specifically, our method achieves the best average AUC results in both pre-training scenarios and fine-tuning scenarios, demonstrating superior generalization ability and the ability to learn features adaptively. Moreover, experimental results against distortion operations (e.g., resizing, Gaussian blur, Gaussian noise, and JPEG compression) show better robustness of our method. In addition, the effectiveness of the key components has been validated through extensive ablation studies.

Although the proposed DFST-UNet can be trained on a relatively small dataset and achieve better performance when testing on the real-world manipulation dataset, the results are still unsatisfactory in practical application. For future work, we will focus on the practical application of forgery localization and create a large-scale and realistic dataset to train the model, which contains forged images continuously collected in real-world scenarios. Moreover, the proposed method re-scaled the input images to a uniform size during preprocessing. However, resizing distorts the forgery artifacts to some extent, leading to performance degradation, especially for high-resolution images. Therefore, we will further develop the model to maintain the original resolution of images and adapt to images of arbitrary size.

## Figures and Tables

**Table 1:** Details of the datasets involved in our experiments. ✓ and ✗ denote whether or not the forgery type is involved.

Dataset	#Image	Forgery Type			Ave. Resolution
		S	C	I	
Columbia [40]	180	✓	✗	✗	892 × 647
COVERAGE [41]	100	✗	✓	✗	476 × 393
CASIA-v1 [42]	920	✓	✓	✗	370 × 270
CASIA-v2 [42]	5123	✓	✓	✗	450 × 344
NIST16 [43]	564	✓	✓	✓	3460 × 2616
IMD2020 [44]	2010	✓	✓	✓	1056 × 849

Abbreviations: S—splicing; C—copy–move; I—inpainting.

**Table 2:** Dataset splits used for training and testing in pre-training and fine-tuning experiments.

Dataset	Pre-Training		Fine-Tuning	
	Train	Test	Train	Test
Columbia [40]	-	180	-	-
COVERAGE [41]	-	100	75	25
CASIA [42]	12,418	920	5123	920
NIST16 [43]	-	564	404	160
IMD2020 [44]	-	2010	1510	500

**Table 3:** Performance comparison using AUC (%) scores under pre-training settings. The top two results are highlighted in bold and underlined, respectively. The symbol “*” indicates our re-implementation using the official public code, and “-” denotes that the result is not available in the literature.

Dataset → Methods ↓	Columbia [40]	COVERAGE [41]	NIST16 [43]	IMD2020 [44]	Average
ManTra-Net [20]	82.4	81.9	79.5	74.8	79.65
SPAN [21]	93.6	**92.2**	84.0	75.0	86.20
MVSS++ [23]	91.3	82.4	83.7	-	85.80
PSCC * [26]	92.7	78.0	81.6	84.8	84.28
DS-UNet * [27]	93.6	80.0	83.8	84.2	85.40
ObjectFormer * [34]	85.0	76.6	81.7	82.2	81.38
ForMoNet [35]	-	85.2	**84.6**	83.9	84.57
Ours	**95.6**	83.7	84.5	**85.5**	**87.33**

**Table 4:** Performance comparison using AUC/F1 scores in % under fine-tuning settings. The top two results are highlighted in bold and underlined, respectively. The symbol “*” indicates our re-implementation using the official public code, and “-” denotes that the result is not available in the literature.

Dataset → Methods ↓	COVERAGE [41]	NIST16 [43]	IMD2020 [44]	Average
RGB-N [19]	81.7/43.7	93.7/72.2	74.8/ -	83.40/57.95
SPAN [21]	93.7/55.8	96.1/58.2	75.0/ -	88.27/57.00
PSCC * [26]	91.7/51.8	99.1/69.0	91.7/46.1	94.17/55.63
DS-UNet * [27]	89.9/31.8	99.8/88.7	92.8/39.8	94.17/53.43
DPMSN [45]	-	98.9/83.3	77.5/29.0	88.20/56.15
TransForensics [33]	88.4/67.4	-	84.8/ -	86.60/67.40
ObjectFormer * [34]	84.5/24.0	97.8/57.1	84.4/19.6	88.90/33.57
ForMoNet [35]	86.2/65.1	95.1/84.7	85.0/43.8	88.77/64.53
Ours	**97.9**/**77.8**	**99.9**/**96.7**	**98.2**/**80.3**	**98.67**/**84.93**

**Table 5:** Robustness comparison results with the AUC (%) metric on the NIST16 [43] dataset under various distortion operations. The best results are highlighted in bold. The “*” denotes our re-implementation using the official public code.

Methods → Distortion ↓	ManTra [20]	SPAN [21]	PSCC * [26]	ObjectFormer * [34]	DS-UNet * [27]	Ours
w/o distortion	78.05	83.95	81.56	81.67	83.82	**84.50**
Resize 0.78×	77.43	83.24	80.66	81.52	84.02	**84.40**
Resize 0.25×	75.52	80.32	75.96	80.52	81.54	**83.59**
Gaussian blur k = 3	77.46	83.10	80.65	81.32	**83.90**	83.27
Gaussian blur k = 15	74.55	79.15	66.36	80.81	**82.17**	81.32
Gaussian noise σ = 3	67.41	75.17	82.27	81.47	82.91	**84.25**
Gaussian noise σ = 15	58.55	67.28	79.09	79.64	81.87	**82.08**
JPEG compression q = 100	77.91	83.59	77.96	81.79	83.36	**84.68**
JPEG compression q = 50	74.38	80.68	75.54	81.48	82.14	**84.32**
Average	72.90	79.07	77.31	81.07	82.74	**83.49**

**Table 6:** Ablation study of different model variants on the Columbia [40] dataset. The results are reported in terms of AUC (%), F1 score (%) and ACC (%). “Ideal” refers to the ideal high-pass filter. “Add” refers to element-wise addition.

Model	RGB	High-Frequency				Fusion		Columbia		
		SRM	Bayar	Ideal	HFEM	Add	HAFM	AUC	F1	ACC
Baseline	✓							91.82	45.76	84.79
Model-v1	✓	✓					✓	87.92	24.95	78.08
Model-v2	✓		✓				✓	88.63	38.16	82.42
Model-v3					✓			93.43	59.51	87.47
Model-v4	✓				✓	✓		91.58	46.24	84.48
Model-v5	✓			✓			✓	95.54	66.34	88.84
Ours	✓				✓		✓	**95.59**	**66.68**	**88.95**

**Note:** Bold font indicates the best performance; ✓ denotes the use of the corresponding method.

**Table 7:** Comparison of model complexity and inference efficiency across different methods.

Model	GFLOPs	Max Memory Usage (MB)	#Parameters (M)	Avg. Inference Time (ms)
PSCC [26]	37.45	1905	**2.75**	23.83
DS-UNet [27]	23.06	**1152**	40.30	38.50
ObjectFormer [34]	65.20	1175	130.57	**19.36**
**Ours**	**11.95**	1200	54.46	21.86

## Data Availability

The datasets used in this study are publicly available online and have been properly cited in the References section. No new datasets were generated during the current study.
